# Optimizing prediction of response to antidepressant medications using machine learning and integrated genetic, clinical, and demographic data

**DOI:** 10.1038/s41398-021-01488-3

**Published:** 2021-07-08

**Authors:** Dekel Taliaz, Amit Spinrad, Ran Barzilay, Zohar Barnett-Itzhaki, Dana Averbuch, Omri Teltsh, Roy Schurr, Sne Darki-Morag, Bernard Lerer

**Affiliations:** 1 Taliaz, Tel Aviv, Israel; 2 Lifespan Brain Institute, The Children’s Hospital of Philadelphia (CHOP) and the University of Pennsylvania School of Medicine, Philadelphia, PA USA; 3 Faculty of Engineering, Ruppin Academic Center, Emek Hefer, Israel; 4 The Dror (Imri) Aloni Center for Health Informatics, Ruppin Academic Center, Emek Hefer, Israel; 5 Biological Psychiatry Laboratory, Hadassah-Hebrew University Medical Center, Jerusalem, Israel

**Keywords:** Depression, Personalized medicine

## Abstract

Major depressive disorder (MDD) is complex and multifactorial, posing a major challenge of tailoring the optimal medication for each patient. Current practice for MDD treatment mainly relies on trial and error, with an estimated 42–53% response rates for antidepressant use. Here, we sought to generate an accurate predictor of response to a panel of antidepressants and optimize treatment selection using a data-driven approach analyzing combinations of genetic, clinical, and demographic factors. We analyzed the response patterns of patients to three antidepressant medications in the Sequenced Treatment Alternatives to Relieve Depression (STAR*D) study, and employed state-of-the-art machine learning (ML) tools to generate a predictive algorithm. To validate our results, we assessed the algorithm’s capacity to predict individualized antidepressant responses on a separate set of 530 patients in STAR*D, consisting of 271 patients in a validation set and 259 patients in the final test set. This assessment yielded an average balanced accuracy rate of 72.3% (SD 8.1) and 70.1% (SD 6.8) across the different medications in the validation and test set, respectively (*p* < 0.01 for all models). To further validate our design scheme, we obtained data from the Pharmacogenomic Research Network Antidepressant Medication Pharmacogenomic Study (PGRN-AMPS) of patients treated with citalopram, and applied the algorithm’s citalopram model. This external validation yielded highly similar results for STAR*D and PGRN-AMPS test sets, with a balanced accuracy of 60.5% and 61.3%, respectively (both *p*’s < 0.01). These findings support the feasibility of using ML algorithms applied to large datasets with genetic, clinical, and demographic features to improve accuracy in antidepressant prescription.

## Introduction

Major depressive disorder (MDD) is a common psychiatric disorder that causes great suffering to patients and their families [1, 2]. The clinical manifestations of MDD are heterogeneous and are composed of multiple symptom domains, leading to situations in which two distinct clinical manifestations may share a common MDD diagnosis with little clinical overlap [3].

Common treatments for MDD include antidepressant medications and psychotherapies. Unfortunately, the current clinical practice of trial and error to determine the optimal treatment for a specific MDD patient lacks efficiency [4]. This inefficiency is plausibly caused, at least in part, by the multifactorial etiology and the above-mentioned phenotypic heterogeneity of MDD [3, 5]. A major gap in the field is tailoring the right treatment for the individual MDD patient (i.e., personalized medicine) [6]. The challenge of predicting which MDD patient will respond to which treatment often results in delayed treatment response, personal suffering, extended disability, higher risk of suicide, and high medical expense [7].

Recent technological advances allow the generation of large amounts of genomic and phenotypic data that pave the way for a new era of brain research, which can hopefully translate to the clinical realm and facilitate the revolution of personalized medicine. To fully leverage the potential of these data, one may utilize the results of decades-long research of factors that were found to be associated with MDD treatment response [8–10], in addition to using sophisticated computational methods and mathematical modeling. Specifically, data-driven analytical approaches and deep analysis of increasingly large databases may now generate new insights into complex clinical challenges, such as optimization of MDD treatment. Machine learning (ML) is an example of such an advanced approach to understanding MDD and its treatment [11]. ML algorithms are broadly viewed as searching through a large space of candidate programs, guided by training experience, to find a program that optimizes the performance metric [12]. The goal of ML in the context of optimizing MDD treatment would be to make predictions about optimal treatment by identifying potentially complex relationships among patients’ genetic, clinical, and demographic data [13]. Indeed, over the past several years a number of research groups have utilized these tools to generate predictions in the context of MDD diagnostics and treatment with impressive results [14], yet these studies have infrequently used a combinatorial approach applied to multimodal data (i.e., data composed of multiple data types) [15].

Here, we used an ML combinatorial approach to generate an algorithm that predicts patient response to antidepressants. To generate and validate this algorithm, we used the large patient datasets from the Sequenced Treatment Alternatives to Relieve Depression (STAR*D) study [16–19] and the Pharmacogenomic Research Network Antidepressant Medication Pharmacogenomic Study (PGRN-AMPS) [20, 21] that include genetic, clinical, and demographic data. We hypothesized that such an application based on integrated multimodal data will enable a more comprehensive and accurate prediction for the treatment of depression and will pave the way for similar analyses of accumulating data by new technologies.

## Materials and methods

### Study participants

#### Main sample

Patient data for algorithm assembly and evaluation were obtained from the STAR*D clinical trial [16–18]. Participants (*N* = 4041) were adult outpatients with nonpsychotic MDD and a score ≥14 on the 17-item Hamilton Depression Rating Scale (Ham-D) [22].

The STAR*D study included four sequential levels of medication or medication combination treatment. Following initiation of treatment, participants were assessed at multiple time points (2, 4, 6, 9, and 12 weeks post-treatment initiation with an optional week-14 visit). Clinical depression severity was measured at each visit by the Quick Inventory of Depressive Symptomatology (QIDS) scale [23], and the Ham-D scale at baseline and exit only. Response to treatment was defined as ≥50% reduction from baseline on either clinical scale. All participants provided written informed consent at enrollment, with consent and study protocols approved by institutional review boards at each participating institution.

For the purpose of the current study, we focused on designing prediction models for citalopram, sertraline, and venlafaxine treatments [18].

#### External sample

Patient data of the PGRN-AMPS [20, 21] were used for replication analysis.

PGRN-AMPS included 529 participants with nonpsychotic MDD and a Ham-D score ≥14 who were recruited at the Mayo Clinic in Rochester, Minnesota, USA. Participants were offered an 8-week course of treatment with either citalopram or escitalopram and depressive symptoms were rated using QIDS and Ham-D scales at initiation, with QIDS being rated at multiple time points afterward (4- and 8-week post-treatment initiation). All patients provided written informed consent. The study protocol was approved by the Mayo Clinic Institutional Review Board. For further details see Ji et al. [21] and Fabbri et al. [24].

For replication analyses purposes, we focused on PGRN-AMPS participants who were treated with citalopram.

### Treatment outcome

We used QIDS score to evaluate participants’ depression level due to the longitudinal use of the QIDS questionnaire throughout all treatment visits, both in the STAR*D study and PGRN-AMPS. Examination of STAR*D response data in level 1 showed that participants who had at least one post-baseline visit had their QIDS score recorded for extensively different periods of treatment: 55.2% of available responses had QIDS records until week 12–14 of the treatment (*n* = 2009, out of 3641), 17.1% until week 9 (*n* = 624), while a substantial portion of participants’ responses, 27.7%, was recorded only until week 6, 4, or even 2 (11.5%, 8%, and 8.2%, respectively). To address these inconsistencies, we generated a response calculation that adjusts for the time of response, named “exponential response” approach, in addition to using the “classic response” approach. These approaches define clinical response as follows:

(i) “Exponential response” approach, whereby an “exponential improvement rate” is calculated per patient, per treatment. The exponential improvement rate is a continuous measure representing exponential fit for the individual longitudinal measurements of QIDS, during a specific treatment. This measure takes into account the change of the score over time, and consequently—the speed and dynamics of the response. The median of the exponential antidepressant improvement rates was calculated independently for each of the STAR*D treatments which were analyzed, using the relevant response data. These median rates were then used to partition the data into two sets, each consisting of half of the participants, creating a dichotomous variable of the exponential antidepressant response (responder/nonresponder), per STAR*D treatment. For PGRN-AMPS citalopram-treated participants, the median citalopram rate from STAR*D was used to partition them into responders and nonresponders.
(ii) “Classic response” approach, where the response is defined by a reduction of at least 50% in the last QIDS score as compared to baseline of each treatment, taking into account a single parameter—change of score from baseline.

All response data were processed using R [25] and Python [26]. Detailed methods for computing the treatment response measures can be found in the Supplementary information.

### Genetic data

A subset of STAR*D participants who were included in the analyses had provided DNA samples for genotyping (*n* = 1953, 48.3% of the overall study participants) [27]. These DNA samples were genotyped on arrays measuring 500,000 or more SNPs that tag the majority of common variants in the human genome.

As part of PGRN-AMPS, DNA from all participants (*n* = 529) was genotyped using blood samples, which were obtained at baseline, measuring approximately the same number of SNPs as for the STAR*D participants.

More details on these DNA samples can be found in the Supplementary information.

### Algorithm assembly and validation

#### Assembly

For algorithm assembly purposes, STAR*D data were used. Following a filtering process of participants’ response data (described in the supplementary information), 1697 remaining STAR*D participants with available genetic, clinical, and demographic data were eventually used for algorithm assembly and validation. These participants were randomly divided into a training set, validation set, and a test set with ~70%:15%:15% ratio, respectively. Due to the fact that some of the participants were treated with two different medications or medication combinations (i.e., proceeded from levels 1 to 2), the training-validation-test division was participant-based throughout the algorithm assembly, rather than level-dependent (e.g., if a participant was randomly assigned to the training group, they remained in this group for all models, treatments, and purposes). These participants’ clinical and demographic enrollment and baseline data, in addition to their available genetic data (in the form of single-nucleotide polymorphisms [SNPs]), were set as ML *features*. The dichotomous exponential response of all participants per treatment was set as the treatment outcome.

We applied a hypothesis-driven approach for the selection of genetic *components* [i.e., genes and microRNAs (miRs)], which were reported to be associated with depression, antidepressant response, metabolism, and side effects. This literature research was conducted by utilizing PubMed and UCSC Genome Browser [28], using compatible search words (e.g., “antidepressant AND genetics”). The overlap of the list of genetic components, which were found during this exploration phase with STAR*D genetic data and the Genome Reference Consortium Human genome build 37 dataset [29], yielded overall 381 genetic components. Common SNPs in and around these mapped components were extracted (up to 6 kb of their flanking regions). Overall, 8120 SNPs were eventually defined as “literature-mapped genetic features.”

Following several processes that included imputation of residual missing data, filtering of features, and features’ encoding processes (e.g., “dummy encoding” [30]), we applied various feature selection algorithms. These included Elastic Net [31] and Least Absolute Shrinkage and Selection Operator [32], and were applied to the data of the training set participants. The feature selection algorithms were submitted initially with over 500,000 features to select from (i.e., the complete STAR*D data)—with only a small minority being “literature-mapped.” This was done in order to make sure the most relevant features for treatment response are characterized and ranked. The above-described literature research was, nevertheless, utilized in several feature selection processes, such that literature-mapped genetic features were given higher weights relative to their non-literature-mapped counterparts.

We then used several subsets of the final selected features to generate several ML models (up to 100 features per model) using the following ML algorithms: support vector machine (SVM) with a linear kernel [33], eXtreme Gradient Boosting (XGBoost) [34], Random Forest [35], and Adaptive Boosting (AdaBoost) [36]. Either 5- or 10-fold repeated cross-validations (CVs) were performed on the training datasets in order to attain optimal parameters, which were then used to re-train the various models using the complete training datasets.

We then generated a single algorithm, which integrates the multiple trained models. This algorithm obtains specific demographic, clinical, and genetic data of a participant and predicts whether they are a “responder” or a “nonresponder” for each of the following medications: citalopram, sertraline, and venlafaxine.

Overall, the data of 1167 STAR*D participants were used in the training phase of the algorithm assembly.

#### Validation

The evaluation of the algorithm performance was done using the validation and final test set of STAR*D participants, which were set aside at the beginning of the algorithm assembly process. These groups consisted of 530 participants overall, of which 271 participants were in the validation set and 259 participants in the final test set. The validation set was used to further edit the algorithm by identifying the best-performing models and subsets of features that were selected during the training phase, while the final test set was not used in any stage of the models’ (and therefore, the algorithm’s) derivation.

For external analysis, the data of PGRN-AMPS participants who were treated with citalopram were used as a second test set. Following a filtering process of participants’ response data, a sample of 132 participants was available for analysis.

The basic patient clinical characteristics and demographics of the various groups are detailed in Supplementary Table 1. For additional information regarding the algorithm assembly and validation, see Supplementary information.

### Statistical analyses

Since some of the validation and test groups, particularly in the case of PGRN-AMPS, were imbalanced in terms of responder/nonresponder ratio (Supplementary Table 2)—balanced accuracy was the main statistical measure that was calculated. Balanced accuracy is defined as the arithmetic mean of the proportions of correct classifications (i.e., accuracies) for each outcome class individually, and therefore balances the accuracy for measuring above-chance generalizability whenever one of the classes is considerably overrepresented in the data [37–39].

To assess statistical significance, a one-sided permutation testing scheme was used to examine how likely the observed balanced accuracies would be obtained by chance (i.e., in a scenario of the absence of real connection between the outcome labels and the final models’ predictive features [40, 41]). For this, 1000 permutation runs were performed for each final predictive model independently. In each permutation run, the outcome labels of the datasets were randomly shuffled, the models were re-tuned via repeated CV on the training set, re-trained with the obtained optimal parameters using the complete training set, and new balanced accuracies were extracted using the trained models’ predictions for the relevant groups (e.g., the final test set). The reported *p* value is the fraction of the 1000 balanced accuracies that were greater than or equal to the balanced accuracy actually observed when the original data were used. Statistical significance was declared for *p* values <0.05.

To examine whether the variances and proportions of the basic patient clinical characteristics and demographics between the various datasets (i.e., training, validation, and the two test sets) are different, we used the Levene’s test [42] for the continuous variables (baseline age, Ham-D, and QIDS scores) and the Pearson’s *χ*^2^ test of independence [43] for the nominal variables (ethnicity, sex).

Additional statistical measures of models’ performance were calculated, including sensitivity, specificity, positive predictive value (PPV) and negative predictive values (NPV), with the latter (PPV and NPV) adjusted in accordance with the fixed prevalence of exponential response in STAR*D (0.5) [44]. Statistical measures of the studies themselves were likewise calculated, including STAR*D’s and PGRN-AMPS’s response rates and null-information rates (NIRs).

In order to extract the estimate of the distribution and confidence interval (CI) of the algorithm’s average balanced accuracy across medications, we used bootstrapping estimation [45]: random resampling with replacement the participants’ test sets 100,000 times, each time generating resampled samples of the same size for each medication independently, for whom the average balanced accuracy across medications was calculated.

### Analyses of the algorithm’s predictive components

For various post hoc analyses solely, the selected features of the final algorithm were segmented into more general distinct components:

- Nongenetic features were segmented into the following domains:
  1. Clinical components, based on clinical diagnosis, physical state, or clinical history (e.g., all features related to the class of anxiety disorders, according to the psychiatric diagnostic symptom questionnaire [46], which assesses psychiatric symptoms in accordance with Diagnostic and Statistical Manual of Mental Disorders, fourth edition, were segmented to “anxiety disorders”).
  2. Demographic components, based on common demographic characteristics (e.g., “age,” “employment”).
- Genetic features (i.e., SNPs) were segmented into functional genetic components according to their location within the genome, either by residing within the gene itself or in an adjacent intergenic region (i.e., intergenic SNPs were mapped to their two most adjacent functional genes, one upstream and one downstream).

Following this segmentation process, we conducted a second literature research focusing on the predictive components of the algorithm in the following manner:

- A search for scientific literature exploring whether the nongenetic components of the algorithm (i.e., clinical and demographic components) are known to be associated with depression or antidepressants. Only if such specific associations were not found, we further examined whether the relevant components were found to be associated with other psychiatric disorders and behavioral or neurological phenotypes.
- A search for scientific literature exploring whether the genetic components, which were not found to be associated with depression or antidepressants in the initial literature research (during feature selection), were possibly found to be associated with other psychiatric disorders and behavioral or neurological phenotypes.

This literature research was done using PubMed, Google Scholar, and PharmGKB [47]. The search words focused on a specific component name along with the relevant keyword (e.g., “ZFPM2 AND psychiatry” or “ZFPM2 AND neurology”).

### Gene Ontology enrichment analysis

For Gene Ontology (GO) enrichment analysis we used Gene Ontology enRIchment anaLysis and visuaLizAtion tool (Gorilla; http://cbl-gorilla.cs.technion.ac.il/) [48], to which the selected algorithm genes (as a target set) and a complete gene list (as a background set) were imported.

For a visual overview of the experimental design please refer to Supplementary Figure 1.

## Results

### Evaluating different approaches to define clinical response to antidepressants

**Fig. 1:**
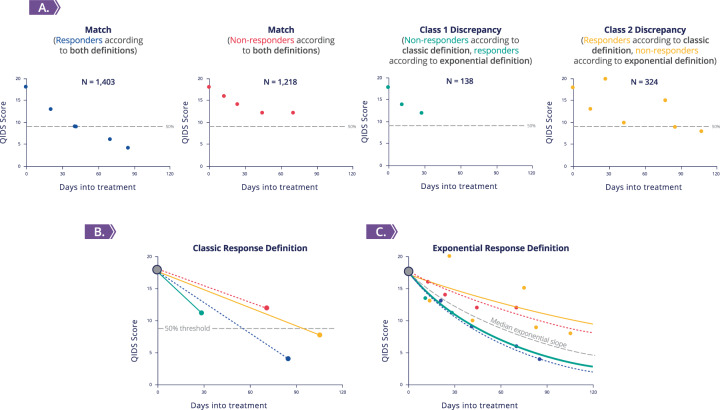
Approaches for defining response to antidepressants. **A** Four representative participants for the three classes of antidepressant response definition approaches’ comparison with their original trajectories: “match” class [blue and red, dotted in (**B**, **C)**] represents 85% of participants, class 1 discrepancy (turquoise) 4.5%, and class 2 discrepancy (yellow) 10.5%; added in *N* are the numbers of participants in each group. **B** Response trajectory for the three classes according to the classic definition of 50% reduction in QIDS score (gray line). **C** Response trajectory for the three classes according to the exponential fit definition; participants with a slope steeper than the median (gray) are categorized as responders (blue and turquoise), and the rest (red and yellow) are categorized as nonresponders. [Both (**B**, **C**) include the time-points that were used in the calculation of the trajectories].

In order to evaluate the optimal way of using the clinical STAR*D data, we first compared the “exponential response” approach to the “classic response” approach in defining response to citalopram treatment (both approaches are described in the Materials and methods section and the Supplementary information). Comparison revealed that the two approaches to define response largely matched (85% agreement, *n* = 2621 out of 3083 citalopram responses’ analyses, Fig. 1 and Supplementary Table 3). However, in 15% of the cases there were discrepancies: in 4.5% of the cases (*n* = 138), participants were labeled nonresponders by the “classic approach” and responders by the “exponential approach” (class 1 discrepancy, turquoise slope in Fig. 1). In 10.5% of the cases (*n* = 324), the “exponential approach” labeled the participants as nonresponders, while the “classic approach” labeled them as responders (class 2 discrepancy, yellow slope in Fig. 1).

Analysis of participants’ responses that are affiliated with class 1 discrepancy shows that these mostly lacked measurement data: the QIDS scores of 77.5% of these responses were not recorded past week 6 (*n* = 107, out of 138, Supplementary Table 3 and Supplementary Figure 2), compared with only 17.8% of all other responses (*n* = 525, out of 2945). Therefore, most of these responses did not have sufficient data over time to reach 50% QIDS score reduction threshold. Notably, this type of discrepancy drops substantially as a participant’s response spans to longer periods of measurements (Supplementary Figure 2). Accordingly, whenever the exponential approach labeled participants who did have their depression level recorded past week 6 as “responders,” they were also labeled as responders by the classic approach (i.e., reached ≥50% reduction in the last QIDS measurement) 97.5% of the time.

Analysis of participants’ responses that are affiliated with class 2 discrepancy showed an opposite trend: 99.4% had sufficient measurement data, i.e.—QIDS records past week 6 (*n* = 322, Supplementary Figure 2), but only 26.5% had over 50% QIDS score reduction by week 9 (*n* = 74, out of 279 with available week 9 scores). Therefore, these responses received lower improvement rates, which labeled the participants as nonresponders by the exponential approach (yellow slope in Fig. 1). In comparison, analyzing the responses of the participant population, which the exponential approach labeled as responders, reveals that 88.7% had over 50% QIDS score reduction by week 9 (980 out of 1105 with available week 9 scores).

### Selecting features for predicting antidepressant response

**Fig. 2:**
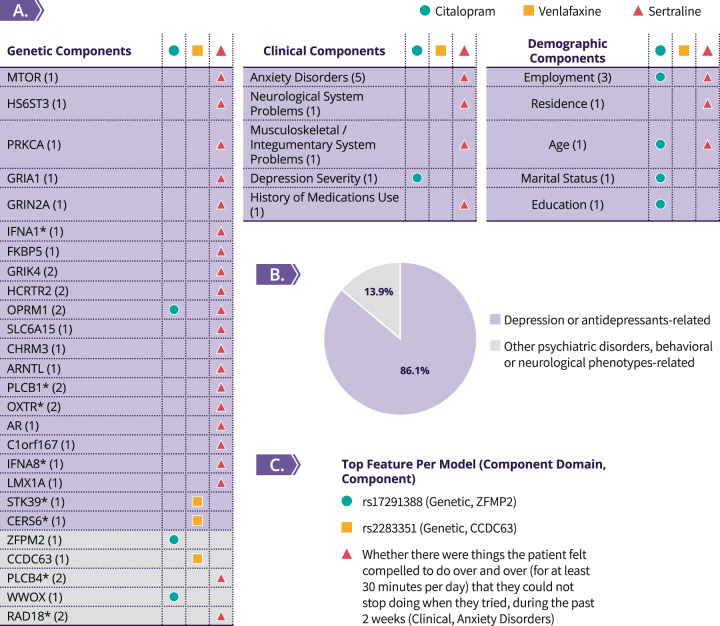
Analysis of the algorithm’s predictive components. **A** List describing the algorithm’s predictive genetic, clinical, and demographic components (within parentheses are the number of features that are mapped to each component, while the asterisks represent components whose mapped genetic features do not reside within the mentioned functional gene itself, but rather in an adjacent intergenic region), with marking per medication. **B** Pie chart describing the distribution of the selected components according to the two classes, following the scientific literature explorations. Only if associations of a component to depression and/or antidepressant were not found, we examined whether it was found to be associated with other psychiatric disorders or neurological phenotypes. **C** Top feature per medication’s final model (according to ROC curve variable importance or absolute coefficient size).

Several feature selection algorithms were applied to the various types of STAR*D data during the training phase, which heavily reduced the number of features (from ~500,000 to 100 or less, per model), followed by a final selection of features that was accomplished using the validation set. The number of features that were ultimately selected and facilitated the generation of the final algorithm’s ML models was therefore narrowed to 43 features (combined). For post-algorithm-assembly analysis purposes, we segmented these 43 selected features into more generalized 36 components of three data domains—genetic, clinical, and demographic (Fig. 2A). Of these 43 features, 27 features are genetic variants that were segmented to 26 genetic components. Notably, a substantial portion of the selected features is nongenetic: nine are clinical features, which were segmented into five distinct clinical components, and seven are demographic features, which were segmented to five distinct demographic components (Fig. 2A).

The post-algorithm-assembly analysis of the group of the selected genetic, clinical, and demographic components showed 86.1% (*n* = 31, out of 36) were previously found to be associated with depression and/or antidepressants (Fig. 2B and Supplementary Table 4). The rest of the components, 13.9% (*n* = 5), were not previously found to be associated with depression or antidepressants, but were found to be associated with other psychiatric disorders and behavioral or neurological phenotypes (e.g., ZFPM2, which was found to be associated with antipsychotic-induced parkinsonism in schizophrenia patients [49]). There were no components that were not previously found to be associated with any psychiatric disorders and behavioral or neurological phenotypes.

Focusing on the 26 selected genetic components of the algorithm, the GO enrichment analysis indicated several distinct types of terms out of the top ten most significantly enriched that were found: 40% are general brain-related terms (e.g., “behavior” or “memory”), additional 40% are neuronal signaling-related terms (e.g., “glutamate receptor signaling pathway”), and the remaining 20% include other terms (e.g., “regulation of body fluid levels,” Supplementary Figure 3A).

### Evaluation of the algorithm performance

The ML models (with the final selected features’ subsets), which achieved optimal results both in the training phase’s CV process and the validation set, were all SVMs with a linear kernel. These models were combined into a single algorithm, which predicted response to antidepressants in the training set (*n* = 1167) with an average balanced accuracy of 73.4% across medications, with the best performance in prediction to venlafaxine (average CV balanced accuracy of 81.2%, SD 11.2, Supplementary Table 5).

In the validation set (*n* = 271), the algorithm predicted response to a medication for a participant with an average balanced accuracy of 72.3% (SD 8.1) across medications (Supplementary Table 6), with the best performance in prediction to venlafaxine (balanced accuracy of 80.2; all balanced accuracies with *p*’s < 0.01). In the final test set (*n* = 259), which was not used in any stage of the algorithm’s derivation, the algorithm achieved average balanced accuracy of 70.1% (SD 6.8) across medications (Table 1), with the best performance in prediction to sertraline (balanced accuracy of 75.5; all balanced accuracies with *p*’s < 0.01). For comparison, STAR*D’s average response rates across medications for the same groups of participants (i.e., validation and test sets) were 55.8% (SD 2.9) and 46.8% (SD 5.3), respectively.

**Table 1:** Algorithm test statistics.

Statistic	Citalopram	Venlafaxine	Sertraline	Mean
Balanced accuracy	60.5%	74.3%	75.5%	70.1% (6.8)
*p* value (balanced accuracy)	<0.001	<0.01	<0.01	–
Sensitivity	67%	70%	69.2%	68.7% (1.3)
Specificity	54%	78.6%	81.8%	71.4% (12.4)
Accuracy	59.8%	75%	75%	69.9% (7.2)
PPV	59.3%	76.6%	79.2%	71.7% (8.8)
NPV	62%	72.4%	72.7%	69% (4.9)

Statistics table describing the success of the algorithm in predicting response and no-response per medication in the final STAR*D test set. *n* = 251 (citalopram), *n* = 24 (venlafaxine), *n* = 24 (sertraline). Standard deviations are given within parentheses.

**Fig. 3:**
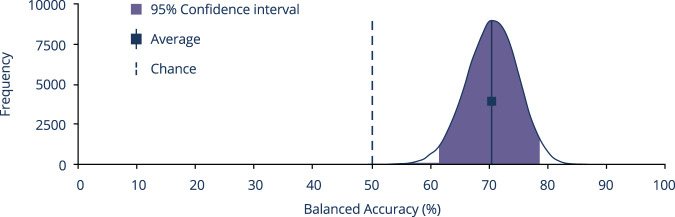
Algorithm’s average balanced accuracy across medications. Bootstrap histogram depicting the results of 100,000 bootstrap runs on the final test set, which generated the bootstrap distribution of balanced accuracies across medications, along with its 95% confidence interval (purple area). The distribution is compared with chance (dashed vertical line) and the observed average balanced accuracy (solid vertical line) in the final test set.

The bootstrap process, which resampled the test’s set participants, yielded a 95% CI of 61.1–78.4% for the algorithm’s average balanced accuracy across medications (Fig. 3).

### Design testing using an external dataset

Next, we analyzed the performance of the algorithm’s citalopram model (Supplementary Table 7) on the PGRN-AMPS sample of 132 citalopram-treated participants, which was skewed in terms of responder/nonresponder ratio (response rate and NIR = 74.2%, Supplementary Table 2) and had significantly different proportions of various ethnicities and sex ratio compared to some of the STAR*D datasets (Supplementary Table 1). The model achieved very similar results to its results in STAR*D (Table 2). Importantly, the balanced accuracy achieved statistical significance in the PGRN-AMPS dataset as well to STAR*D.

**Table 2:** Citalopram model statistics for both studies’ final test sets: statistics table describing the success of the citalopram model in predicting response and no-response for citalopram-treated participants, in STAR*D and PGRN-AMPS final test datasets.

Statistic	STAR*D	PGRN-AMPS
Balanced accuracy	60.5%	61.3%
*p* value (balanced accuracy)	<0.001	<0.01
Sensitivity	67%	75.5%
Specificity	54%	47.1%
Accuracy	59.8%	68.2%
PPV	59.3%	58.8%
NPV	62%	65.8%

*n* = 251 (STAR*D), *n* = 132 (PGRN-AMPS).

## Discussion

We present the assembly process of an algorithm that predicts individual response to antidepressants, and its performance on validation and testing datasets. The algorithm demonstrates its capabilities of selecting a suitable antidepressant for an individual patient with an average balanced accuracy of 70.1% in a final test set, compared to 46.8% average initial response rate in the same set of STAR*D participants. This finding is of high clinical importance, as clinical experience and evidence show that current methods of determining the optimal treatment for MDD are limited and driven by trial and error [50]. Specifically, evidence emerging from the STAR*D study indicates that it took four controlled treatment steps, lasting 12–14 weeks each, to achieve an overall cumulative response rate of 74% [4, 18]. Notably, the modeled performance of the algorithm using STAR*D data suggests that a comparable response rate can potentially be achieved on the initial treatment selection.

Designing a clinically informative algorithm for medication selection requires a comprehensive definition of treatment response, to allow accurate classification of participants’ outcomes. Therefore, we presented in this study a novel exponential approach to the definition of antidepressant response, taking into account the change of symptoms load (i.e., clinical scores) over time, and consequently—the speed and dynamics of the response. By doing so, this approach normalizes for varying temporal patterns of measurements for different participants. This normalization is implemented by factoring multiple longitudinal measurements, which were made possible due to the deep longitudinal phenotyping of STAR*D participants, as well to PGRN-AMPS participants to some extent. These intra-treatment measurements are mostly ignored in the traditional definitions of response, which often rely on an endpoint measurement compared to baseline [51]. We argue that disregarding data collected at multiple time points be a shortcoming, considering that it was repeatedly shown that trajectories of antidepressant response are commonly not linear [52–56], with some studies stating that early therapeutic effects may be better predictors of a subsequent positive long-term outcome [55–57]. Therefore, we hypothesize that this approach may be more sensitive to determine treatment success. We suggest that using such exponential methods to define response in other datasets merits further investigation.

On average, the efficacy of antidepressants is in the range of 42–53% [58, 59], using the classic definition of response (a reduction of ≥50% in the last score as compared to baseline). In comparison, current available pharmacogenomic tests, which rely on the genetic background to tailor drug prescription, may yield slightly higher response rates (39–64%) [60]. Therefore, we chose to include genetic data in our predictive algorithm. The GO enrichment analysis, which was performed on the algorithm’s genetic predictive components, revealed several significant neuronal signaling-related terms. This is not surprising, since many genes that were reported in the scientific literature as associated with depression and antidepressants’ response (and were therefore collected in the literature search phase of our feature selection process) are largely based on studies that focused on neuronal signaling pathways. Nonetheless, the significant improvement in treatment success rates provided by the input of these genes reinforces the notion whereby in-depth knowledge of an individual’s neurotransmitter circuit connections may lead to better treatment selection for depression [61, 62].

It is likely that genetic variation among individuals, despite its significant contributions, is only one of many individual factors that influence the chance of achieving a response to antidepressants [9, 11]. Notably, heritability in depression is estimated to be 37% [63]. This suggests that, although important, genetic variation can only partly reveal the complex individual difference in depression pathobiology, and might also suggest that genetics will only partly explain treatment response [64, 65]. Such nongenetic basis is exemplified by previous works of research groups, which used ML algorithms applied on clinical and demographic data exclusively, to achieve significant success rates for the prediction of depression treatment outcomes [66, 67].

Introduction of ML-based prediction tools that aspire to have clinical relevance should aim to increase generalizability, usually through validation in completely new and independent datasets [68]. Therefore, we sought to examine our analytical design in an external sample of MDD patients, the PGRN-AMPS dataset, using the citalopram model, which used features that were attainable from both studies. The resemblance and statistical significance of this model’s success rates for citalopram-treated participants in STAR*D and the PGRN-AMPS dataset were therefore highly encouraging, and support the generalizability of our algorithm to other patients’ samples. However, these results have limitations: this evaluation was done only with the citalopram model, a single model out of three final algorithm’s models, which also originally had lower success rates in comparison to the other medications’ final models. In addition, the PGRN-AMPS dataset has significantly different proportions of ethnicities compared to all STAR*D datasets, and a significantly different sex ratio compared to the STAR*D training set (both variables are not used as predictive features by the final algorithm). Further, the citalopram model’s accuracy in the PGRN-AMPS sample did not surpass its unusually high NIR, which results from the overall study’s unique response rate, which was previously reported [20]. Still, when we used balanced accuracy (a measure that overcomes the differences in response rates between studies [37–39]), our analytical design achieved comparable predictive capacities in the PGRN-AMPS sample as observed in the STAR*D dataset.

The algorithm presented here might serve as a basis for clinical support platforms, which are part of the emerging field of precision psychiatry, i.e.—the approach for psychiatric treatment and prevention that takes into account each person’s variability in genes, environment, and lifestyle [69–72]. These tools are expected to significantly improve with time as data derived from next-generation sequencing technologies and electronic health records are accumulating and are becoming increasingly available. Future applications in this field, and specifically with depressive disorders, can include the formulation of better diagnostic and prognostic techniques, as well as a better understanding of the neural circuits involved in their etiology [73].

Several limitations of our study should be acknowledged. First, the algorithm was developed from a training set derived from a single study (although the largest and most comprehensive to date), STAR*D. However, the fact that we found one of our generated models to perform similarly well in a totally different sample than the one we used for its training phase mitigates some of the concerns regarding the generalizability of the findings. In addition, the available STAR*D SNP data did not include sufficient information to enable metabolizer phenotypes’ inference, which could potentially elevate the presented algorithm’s success rates, considering the abundant evidence in regards to the metabolism status effects on medication response [74, 75]. In addition, the algorithm could only create predictions for three medications used in STAR*D, whereas clinicians these days have more therapeutic options that were not included in our analyses. Notably, our findings do suggest that in two different classes of antidepressants (i.e., SSRI and SNRI)—the algorithm performed better than clinically expected. Lastly, we assessed the algorithm performance retrospectively; prospective future studies are needed to further solidify the evidence presented in this study [68].

## Conclusion

In summary, there is a need for new approaches to help clinicians improve the treatment of depression and other psychiatric disorders. Applying ML approaches to genetic, clinical, and demographic data is a promising method to achieve this goal. The challenge of any prediction algorithm is to select the right combination of features that will predict a well-defined clinical outcome. The algorithm that we describe here may be used as a tool to tackle some of these challenges and support clinicians’ decisions, aiding in a more precise choice of antidepressant medication. Moreover, utilizing some of the genetic factors that are found to increase the prediction accuracy of patients’ response to antidepressants could potentially allow a better understanding of medications’ mechanism of action, and may lead to the identification of novel molecular targets, consequently driving the development of novel treatments for depressive disorders.

## Supplementary information

Supplemental Information

## Data Availability

The computer code is available from the authors upon reasonable request.
