# Supplementary material for: Optimizing prediction of response to antidepressant medications using machine learning and integrated genetic, clinical, and demographic data
Source: Transl Psychiatry. 2021 Jul 8;11:381. doi: 10.1038/s41398-021-01488-3 (PMC8266902; doi:10.1038/s41398-021-01488-3)
Supplement: Supplementary file 1 — Supplemental Information [file 41398_2021_1488_MOESM1_ESM.docx]

**Supplementary**

**Antidepressant response calculations**

50% QIDS score reduction (i.e., “classic antidepressant response”): To calculate this measure, scores from the participant’s last day of treatment were subtracted from the participant’s initial score of the same treatment, and then divided by this initial score, irrespective of how many weeks they were in treatment. If the resulting change of score was greater than or equal to 0.5, the participant was labeled as a “responder” for this specific treatment. If the participant had a change in score less than 0.5, the participant was labeled as a “non-responder” for this specific treatment.

Exponential fit for the personal measurements of QIDS (i.e., “exponential antidepressant response”): To calculate this measure, each participant’s QIDS scores during a specific treatment were plotted against the number of days they were in treatment. At first, linear regression model was fitted to the data. Due to the linear regression model’s lack of fit for most participants (data not shown), the linear model was applied to an exponential model, which is simply a linear fit in logarithmic scale. The y-intercept, *a*, and the slope, *b*, derived from the linear equation - were input to the exponential equation:

$$g\left( x \right)= e^{a+bx}$$

A “*consecutive days’ decrease in depression constant*” for each participant per treatment was calculated as follows:

$$\frac{g(t+1)}{g(t)}= \frac{e^{a+b(t+1)}}{e^{a+bt}}=\frac{e^{a}*e^{bt}*e^{b}}{e^{a}*e^{bt}}=e^{b}$$

Where *t* is any specific day during the treatment, and *t + 1* is the following day. Hence, $e^{b}$ represents the ratio of the depression score between any two consecutive days of a participant’s treatment according to the exponential model. Lower values of the $e^{b}$ constant mean earlier decrease in QIDS score. Therefore, its multiplicative inverse $\frac{1}{e^{b}}$ means the opposite: as this variable increase, the response to the antidepressant is earlier, in terms of changing QIDS score. Thus, $\frac{1}{e^{b}}$ was labeled as the “*exponential improvement rate*”. Participants whose exponential improvement rates were higher than the STAR*D median exponential improvement rate had experienced an earlier decrease in QIDS score than participants whose exponential improvement rates were lower than the STAR*D median, per STAR*D treatment’s median exponential improvement rate. Hence, the formers were labeled as treatment “responders” for the treatment at hand, and the latter as treatment “non-responders”.

Treatment data that could not be modeled due to an excessive number of data gaps or irregular score pattern were filtered from both datasets. Additionally, 5% of the STAR*D modeled treatment data with the highest sum of squares error were filtered as well.

**Data access**

STAR*D

NIH-supported “Sequenced Treatment Alternatives to Relieve Depression” (STAR*D) data were obtained through a limited access data use certificate (DUC), awarded to Prof. Bernard Lerer. Access to STAR*D data and biospecimens is available to qualified investigators by application to the NIMH Repository and Genomics Resource through submission of a Request Access Form (<https://www.nimhgenetics.org/request-access/how-to-request-access>).

PGRN-AMPS

NIH-supported “Pharmacogenomics Research Network Antidepressant Medication Pharmacogenomic Study” (PGRN-AMPS) data were obtained through a limited access data use certificate (DUC), awarded to Dr. Dekel Taliaz. Access to PGRN-AMPS data is available to qualified investigators by application to the Database of Genotypes and Phenotypes (dbGaP) Authorized Access System through submission of a Data Access Request (<https://dbgap.ncbi.nlm.nih.gov/aa/wga.cgi?page=login>).

**Genetic data**

STAR*D

A subset of participants who were included in the analyses had provided DNA samples for genotyping (n = 1,953 , 48.3% of the overall study participants), as described in earlier reports^1^. DNA was extracted from blood or lymphoblastoid cell lines and genotyped on arrays measuring 500,000 or more single-nucleotide polymorphisms (SNPs) that tag the majority of common variants in the human genome. DNA samples were then genotyped using the Affymetrix© Human Mapping 500K Array and the Genome-Wide Human SNP Array 5.0 (Affymetrix, Santa Clara, California, USA) with >=99% concordance observed in the 12 samples genotyped on both platforms. Genotyping and quality-control methods were previously described^2^. These STAR*D genotypes were made available to us through the National Institute of Mental Health Human Genetic Initiative (<https://www.nimhgenetics.org/>) through limited access data use certificates, as previously detailed.

PGRN-AMPS

DNA from all participants (n = 529) was genotyped using blood samples which were obtained at baseline, at the RIKEN Center for Genomic Medicine (Yokohama, Japan) using Illumina human 610-Quad BeadChips (Illumina, San Diego, California, USA), as previously described^3–5^. Quality control assessments included overall genotype concordance rates based on duplicate sample genotyping and Mendelian inheritance checks based on genotyping of a CEPH trio of two parents and their child. These PGRN-AMPS genotypes were made available to us through the National Institutes of Health and the Database of Genotypes and Phenotypes (dbGaP) Authorized Access System (<https://dbgap.ncbi.nlm.nih.gov/>) through limited access data use certificates, as previously detailed.

**Additional algorithm assembly & validation information**

STAR*D SNPs were extracted, imputed for residual missing data and filtered using PLINK^6^ and Beagle^7^, via analysis of Hardy-Weinberg Equilibrium (p-value <= 2.022 e-18), minor allele frequency (<= 0.02), original missingness rate (>=10%), batch effect bias test (after Bonferroni correction with p-value <= 0.05), and linkage disequilibrium (a sliding window of 50 SNPs, step of 5 SNPs, and pairwise r^2^ threshold of 0.95). The three selected SNPs of the final algorithm’s citalopram model were fully imputed for the PGRN-AMPS dataset using PLINK and Beagle (all with high imputation quality; allelic r^2^ of 0.87-0.97). The database of the 1000 genomes project was used as a reference panel for these analyses^8,9^.

Demographic and clinical enrollment and baseline features were filtered by removing features with excessive missing values (>10%), and near-zero variance, as well as imputation of residual missing data using the VIM package in R (kNN approach)^10^.

Of the final selected features, there were 2 pairs of genetic features which were found to be in some linkage disequilibrium (>0.1 pariwise-r^2^ value) – one pair with r^2^ value of 0.71, and the second with r^2^ value of 0.6, both pairs were in the basis of the final sertraline model generation. While in GWAS analyses it is important to set higher thresholds of linkage disequilibrium, both for reducing computational cost, and more importantly – in order to be able to deduce direct or indirect (i.e., causation) effect of a SNP on an examined phenotype^11,12^, this is not precisely the case for some ML models, such in the case of SVMs^13,14^. This is since SVMs’ regularization parameter may alleviate, at least in part, the problem of multicollinearity of features, and therefore – they may perform well in the presence of LD^13,14^.

**Supplementary Table 1.** Basic patient demographics & clinical characteristics of the various datasets used in the study. Significant differences between the variances and proportions of these data were found for ethnicity (PGRN-AMPS test set vs. all other (STAR*D) sets, all *p*’s <0.001) and for sex (PGRN-AMPS test set vs. the training set and the validation set vs. the training set, both *p*’s <0.05); both variables are not part of the predictive features’ set used by the final algorithm.

**
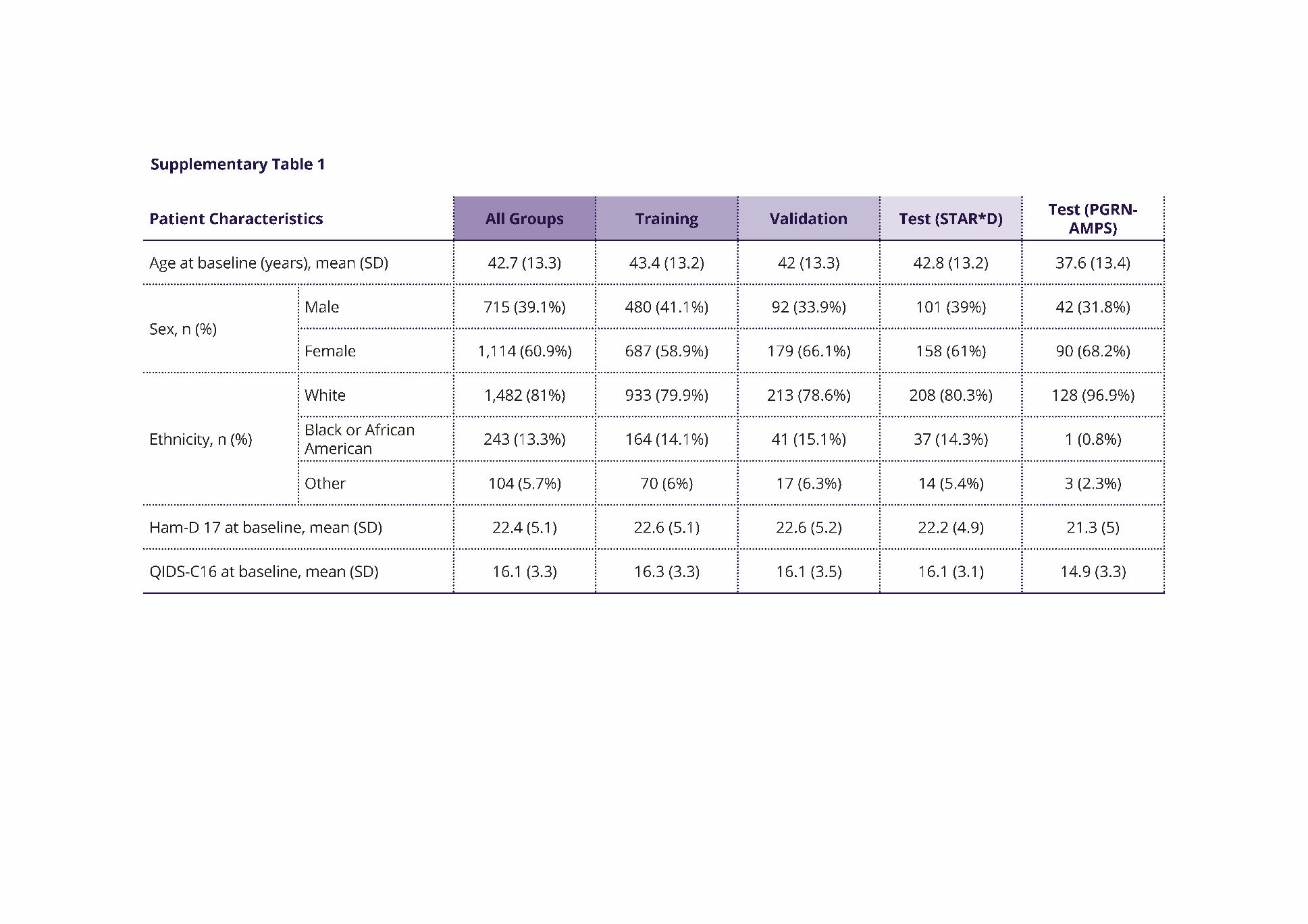
**

**Supplementary Table 2.** Response rate (i.e., percentage of responders out of the total number of participants), per medication and per participants’ set used in the study, according to the exponential response definition.


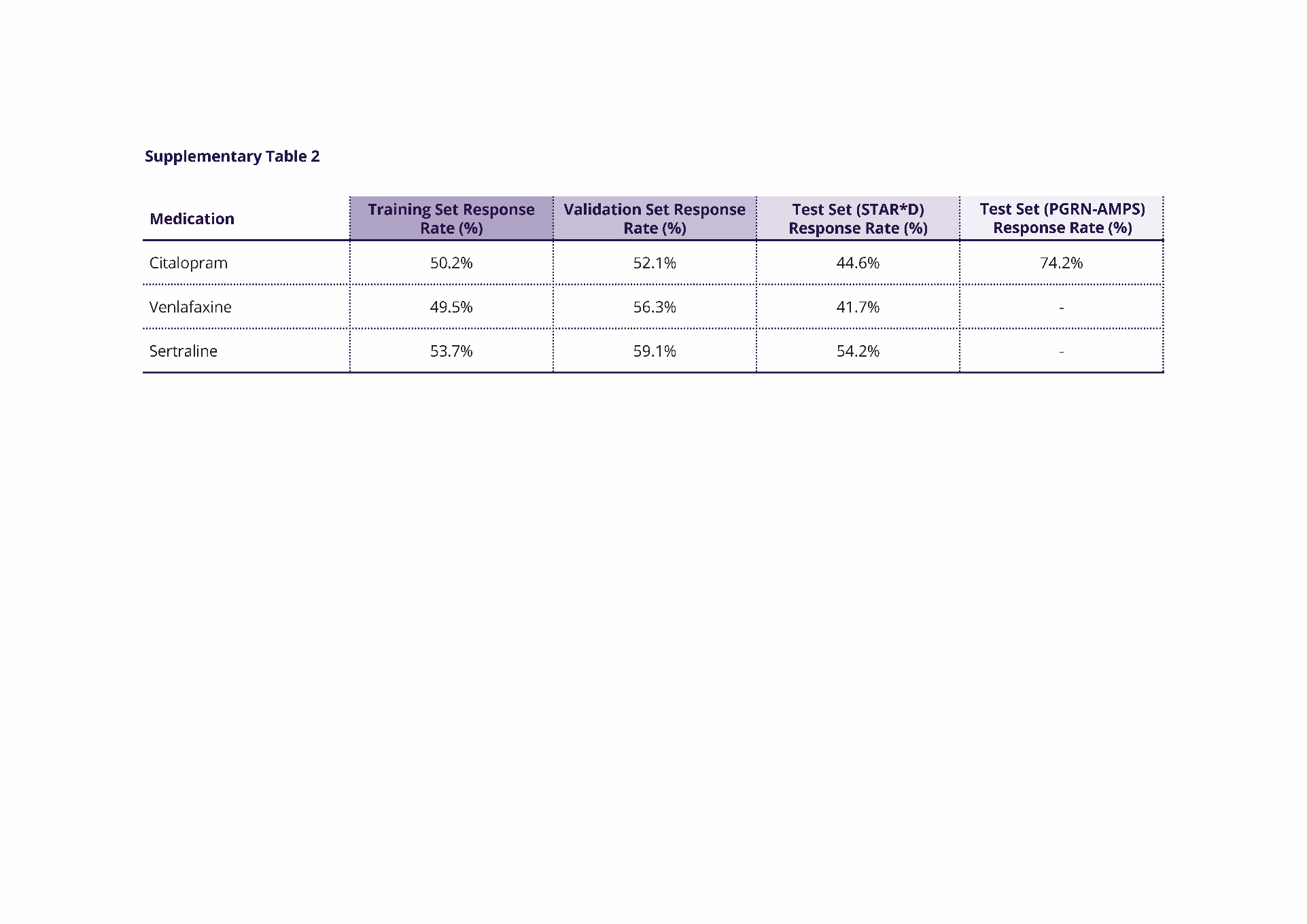


**Supplementary Table 3.** STAR*D measurements of QIDS score and comparisons between the two response definition approaches, using citalopram treatment as an exemplar. N (Responses) = the amount of participants’ responses whose last QIDS score was measured in week (i) of treatment. Response Rate = percentage of “responders” out of all response analyses under the relevant “last week (i)” column. Match (Responders) = percentage of participants defined as “responders” both by the exponential approach and the classic approach. Match (Non-responders) = percentage of participants defined as “non-responders” both by the exponential approach and the classic approach. Class 1 Discrepancy = percentage of participants defined as “responders” by the exponential approach and as “non-responders” by the classic approach. Class 2 Discrepancy = percentage of participants defined as “non-responders” by the exponential approach and as “responders” by the classic approach.


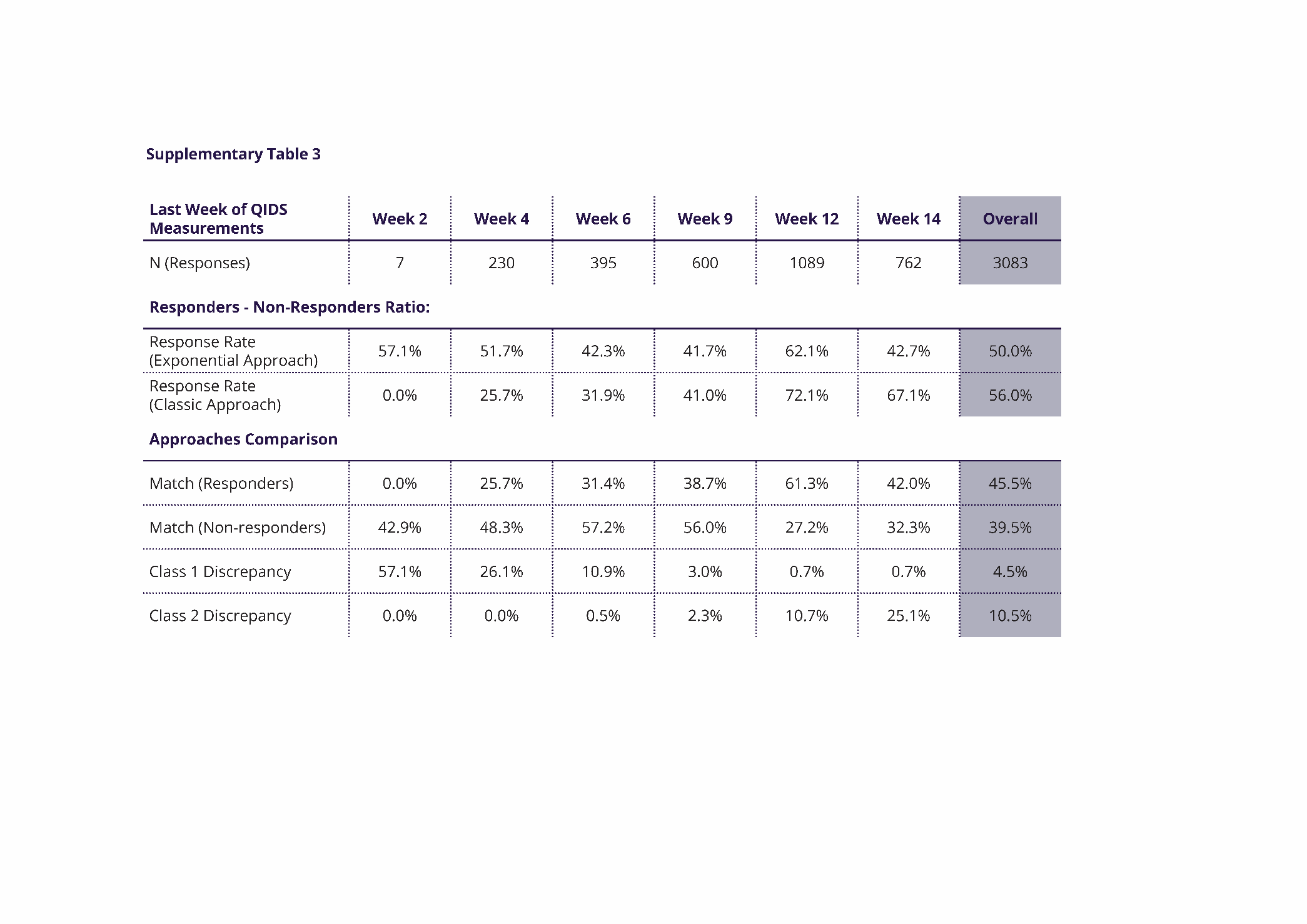


**Supplementary Table 4.** Algorithm selected components’ literature search results. The table shows the components for which scientific literature in the context of association with depression and/or antidepressant was found.


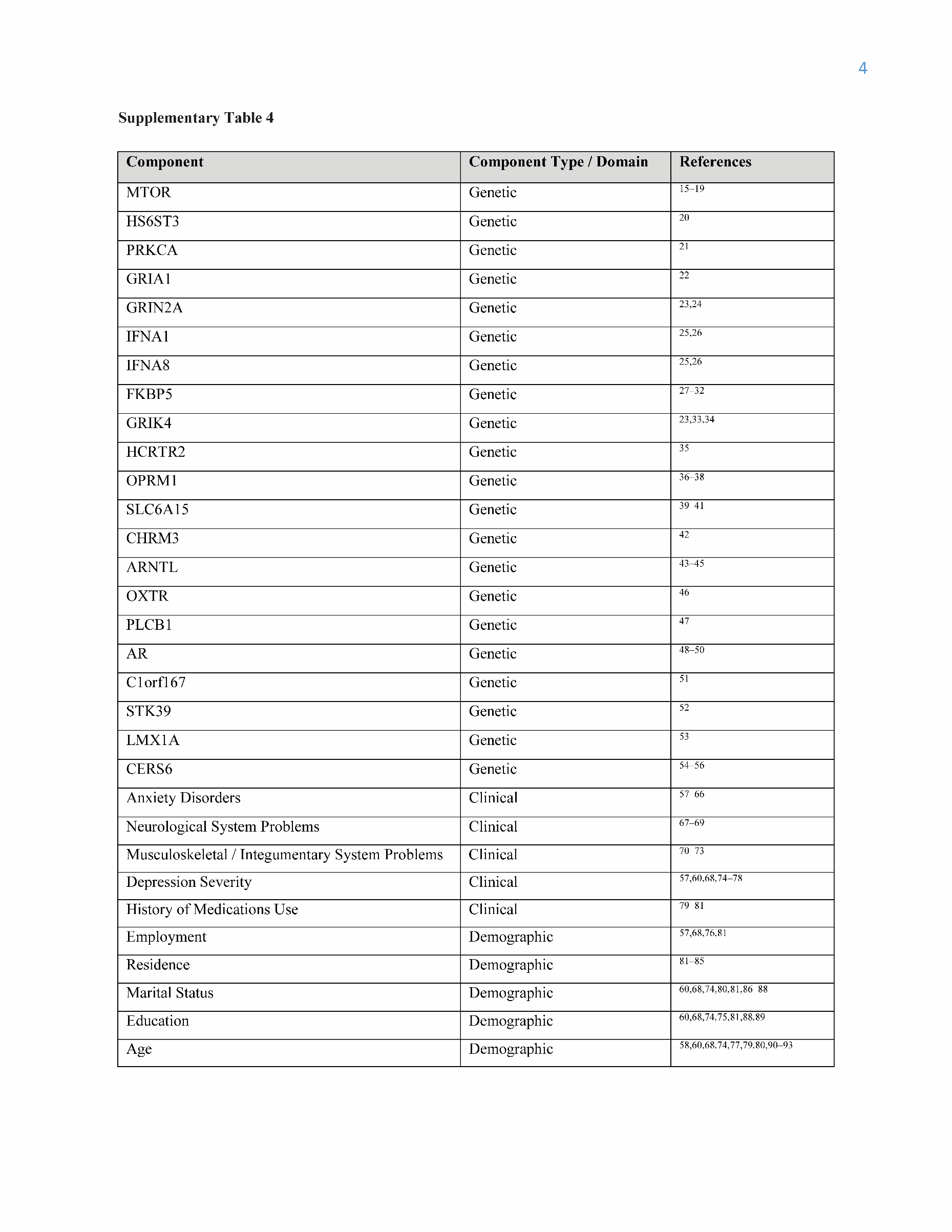


**Supplementary Table 5**. Algorithm cross-validation statistics. Statistics table describing the success of the algorithm in predicting response & no-response per medication, in the cross-validation process of its training phase. n = 1,146 (citalopram), n = 95 (venlafaxine), n = 67 (sertraline). Results under the different medications are cross-validation averages across all holdout folds; standard deviations in parentheses.


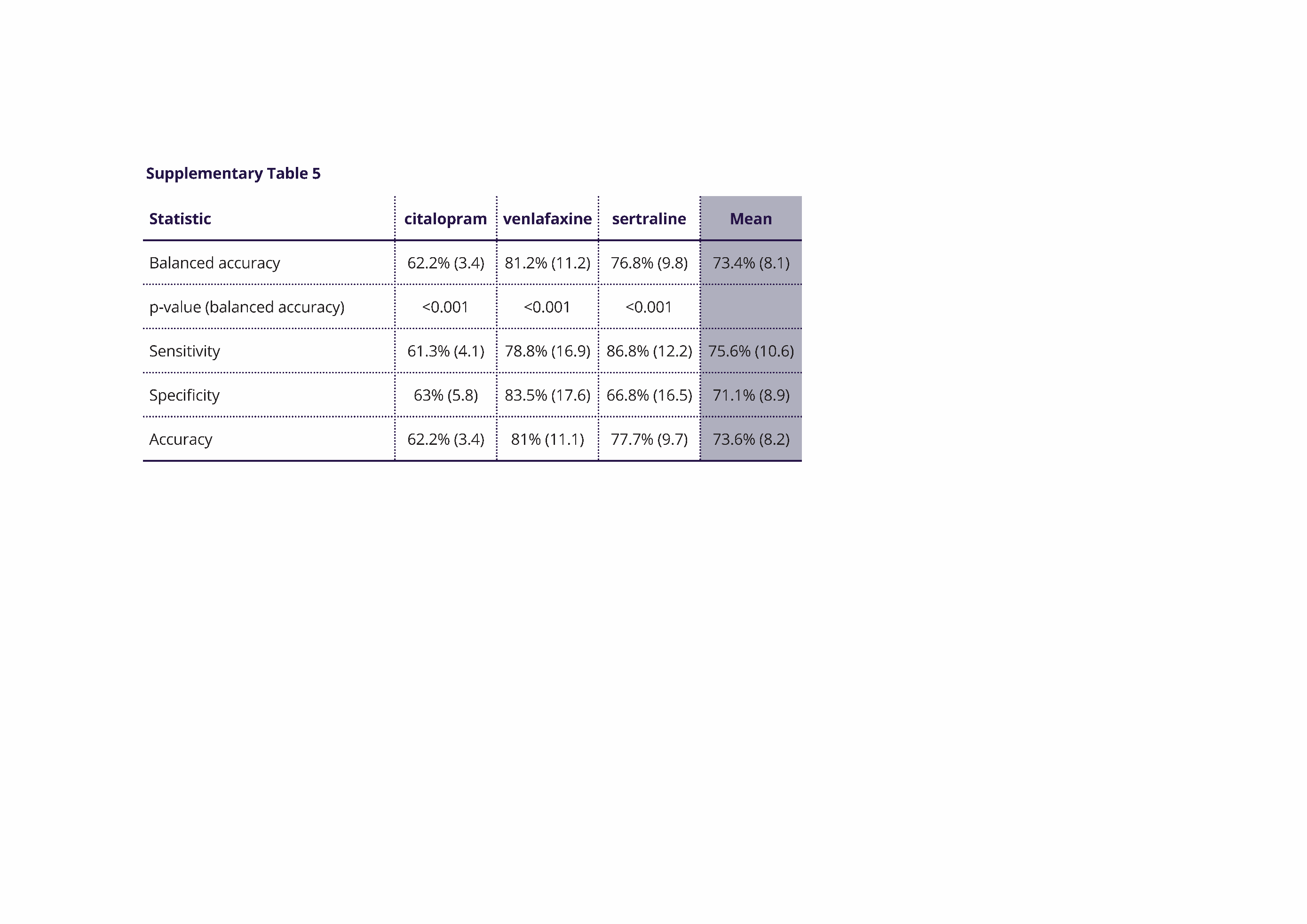


**Supplementary Table 6.** Algorithm validation statistics. Statistics table describing the success of the algorithm in predicting response & no-response per medication in the STAR*D validation set. n = 259 (citalopram), n = 16 (venlafaxine), n = 22 (sertraline). Standard deviations in parentheses.


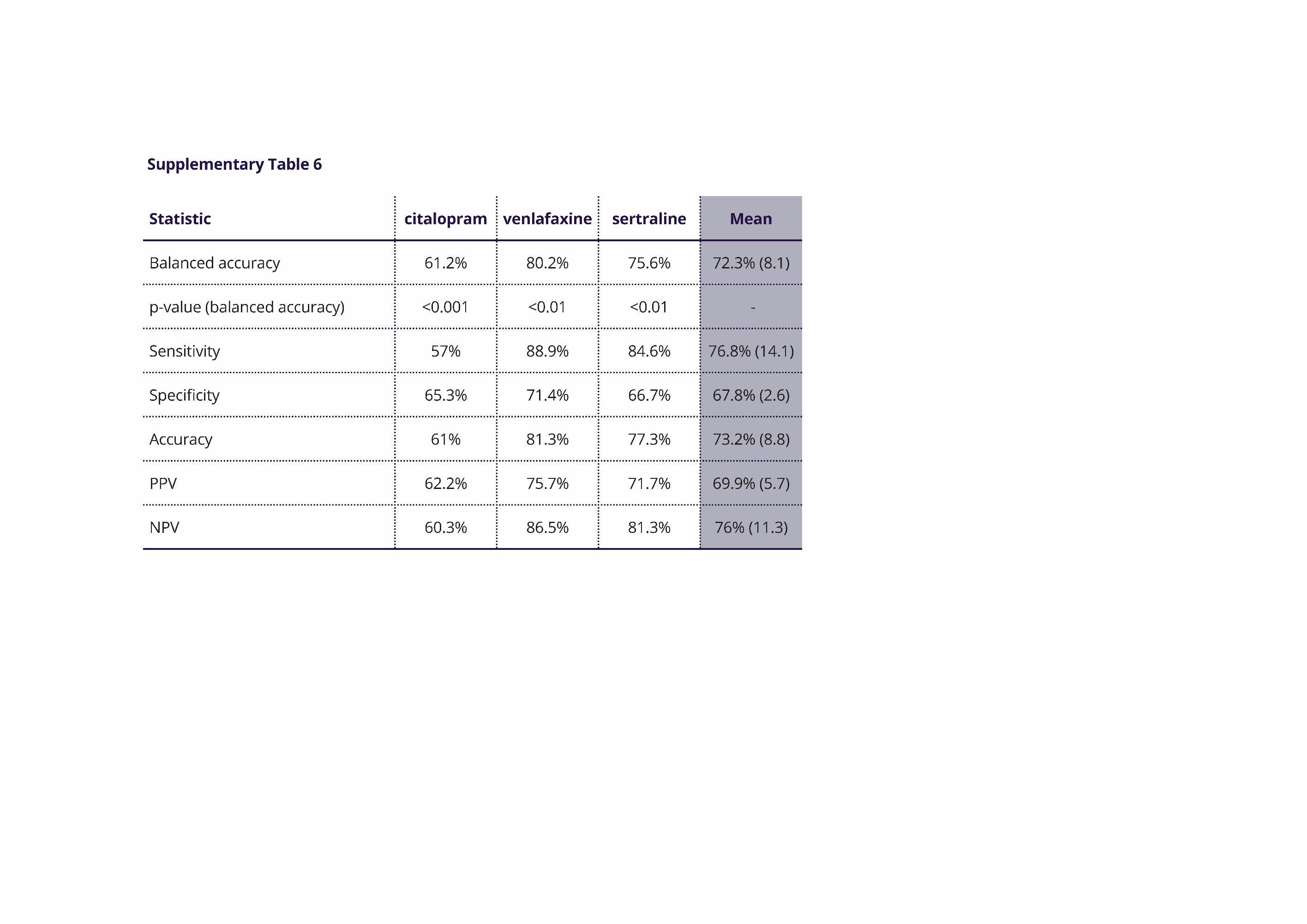


**Supplementary Table 7.** Citalopram model description: the citalopram model’s predictive components and their mapped features.


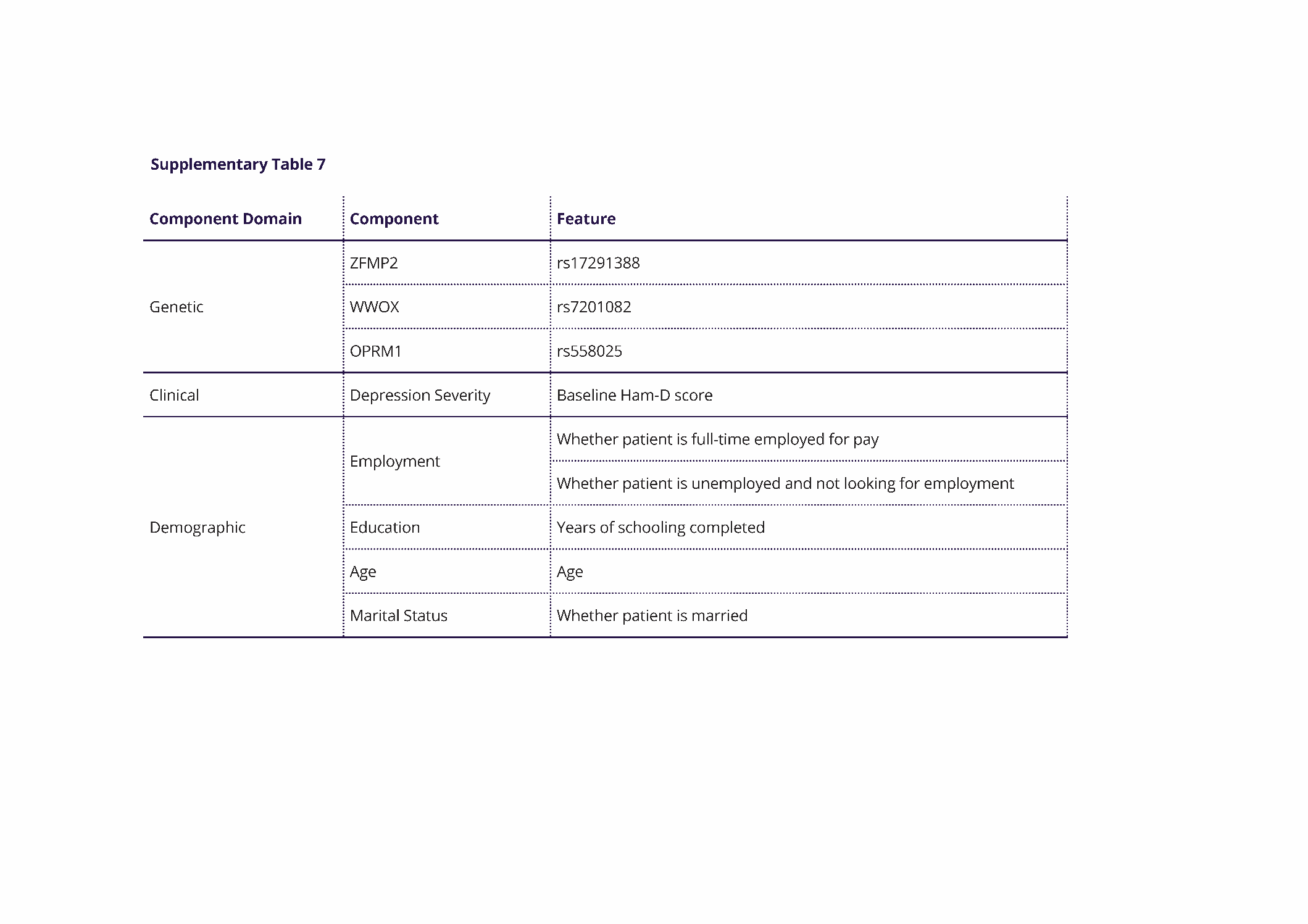


**Supplementary Figure 1.** Study design: the process of generating and testing the predictive algorithm.


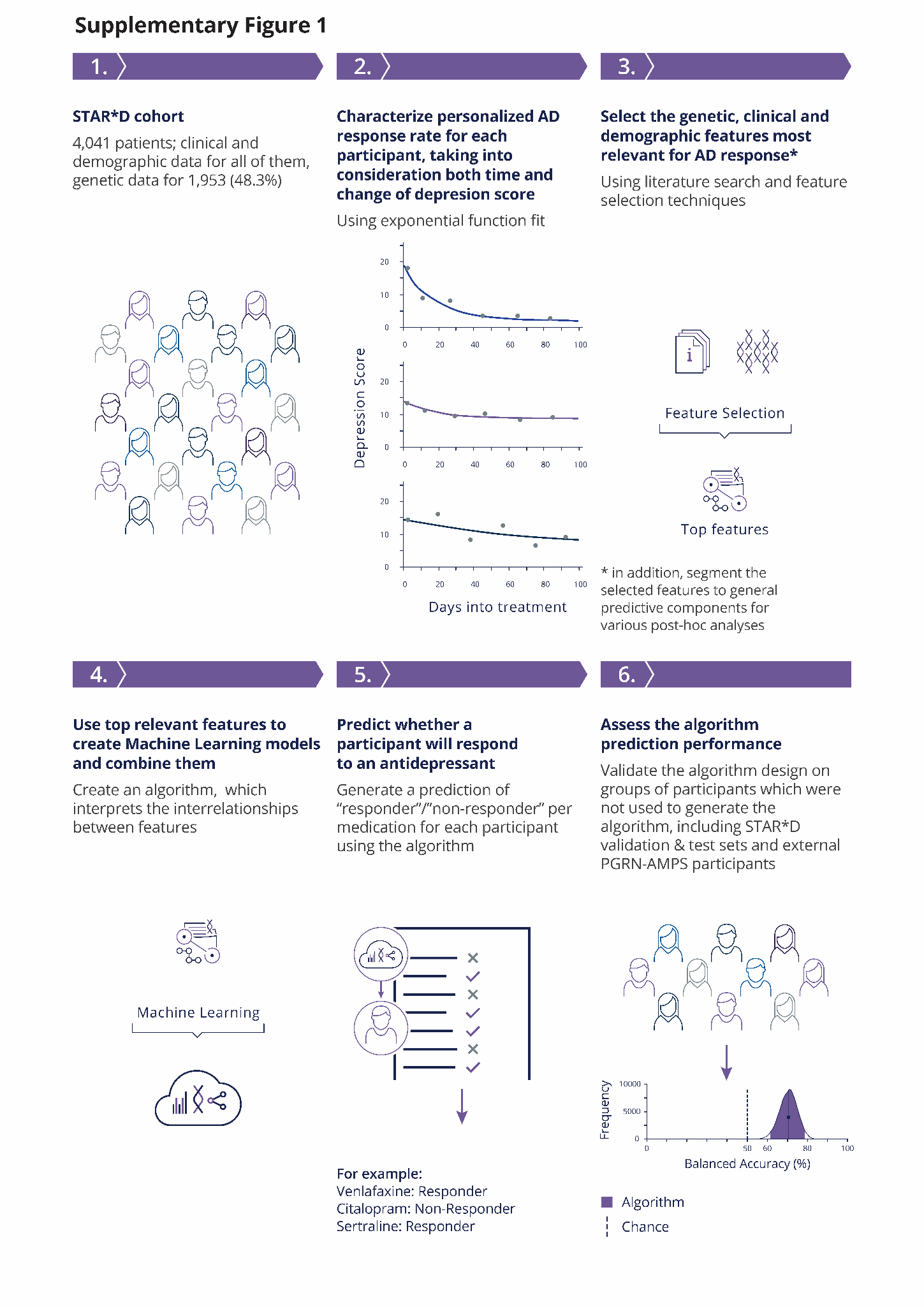


**Supplementary Figure 2.** Dynamics of the three classes comparing the two approaches of response definition (exponential approach vs. classic approach), using citalopram responses as an exemplar. Match = responses with matching exponential response and classic response classification. Class 1 Discrepancy = responses with exponential classification of “responder” and classic classification of “non-responder”; Class 2 Discrepancy (%) = responses with exponential classification of “non-responder” and classic classification of “responder”.


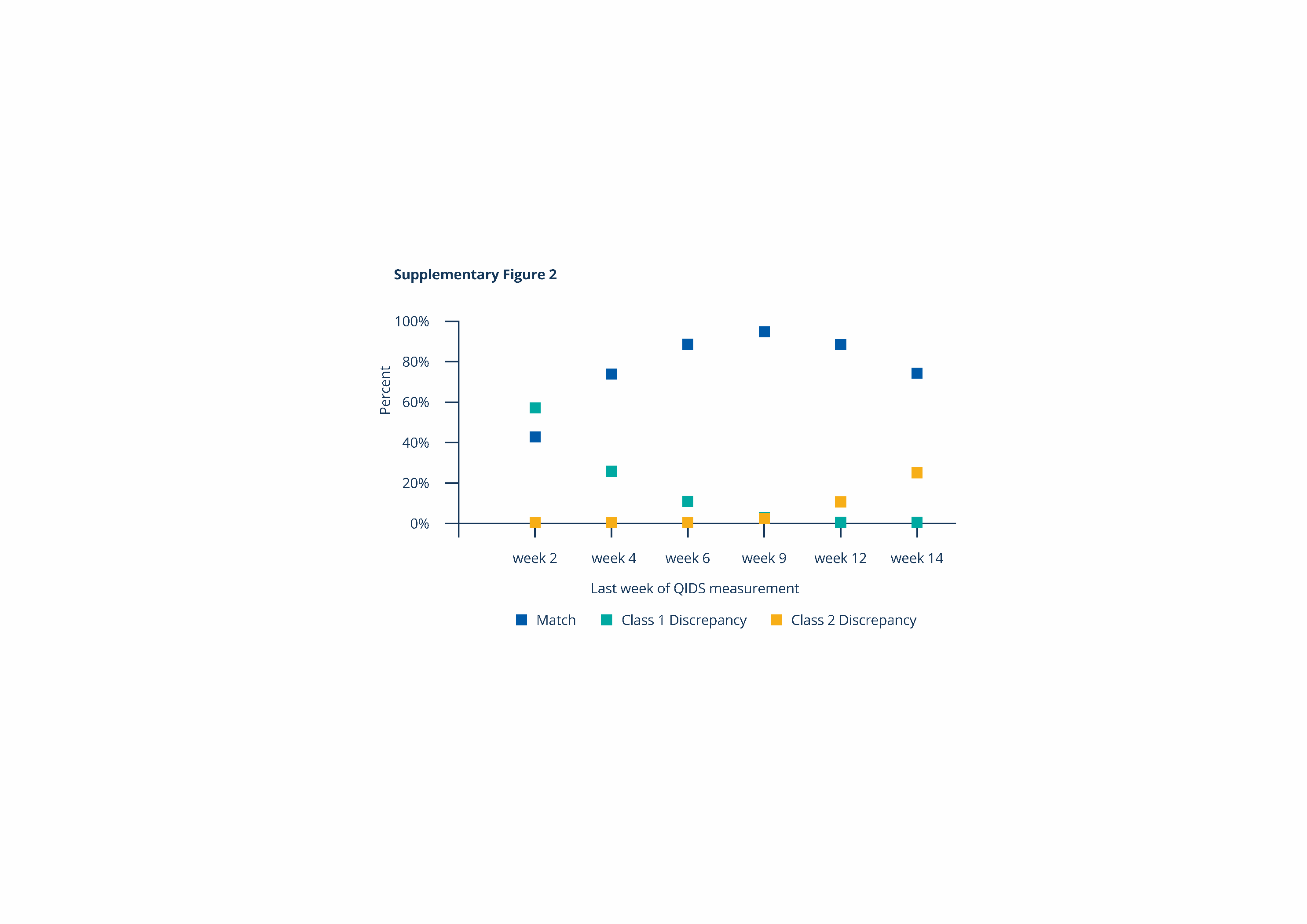


**Supplementary Figure 3.** Gene Ontology (GO) enrichment analysis of the algorithm selected genes. **a.** Bars representing -log10 (p-value) of enrichment computed in accordance with the HG model. Blue bars represent GO terms which are related to general brain functions, green bars represent GO terms which are related to neuronal signaling, and grey bars represent other GO terms (not belonging to each of the aforementioned groups). **b.** List of genes found in relation to neuronal signaling-related terms (green bars), n = 9 (34.6% out of the 26 algorithm selected genes).

**
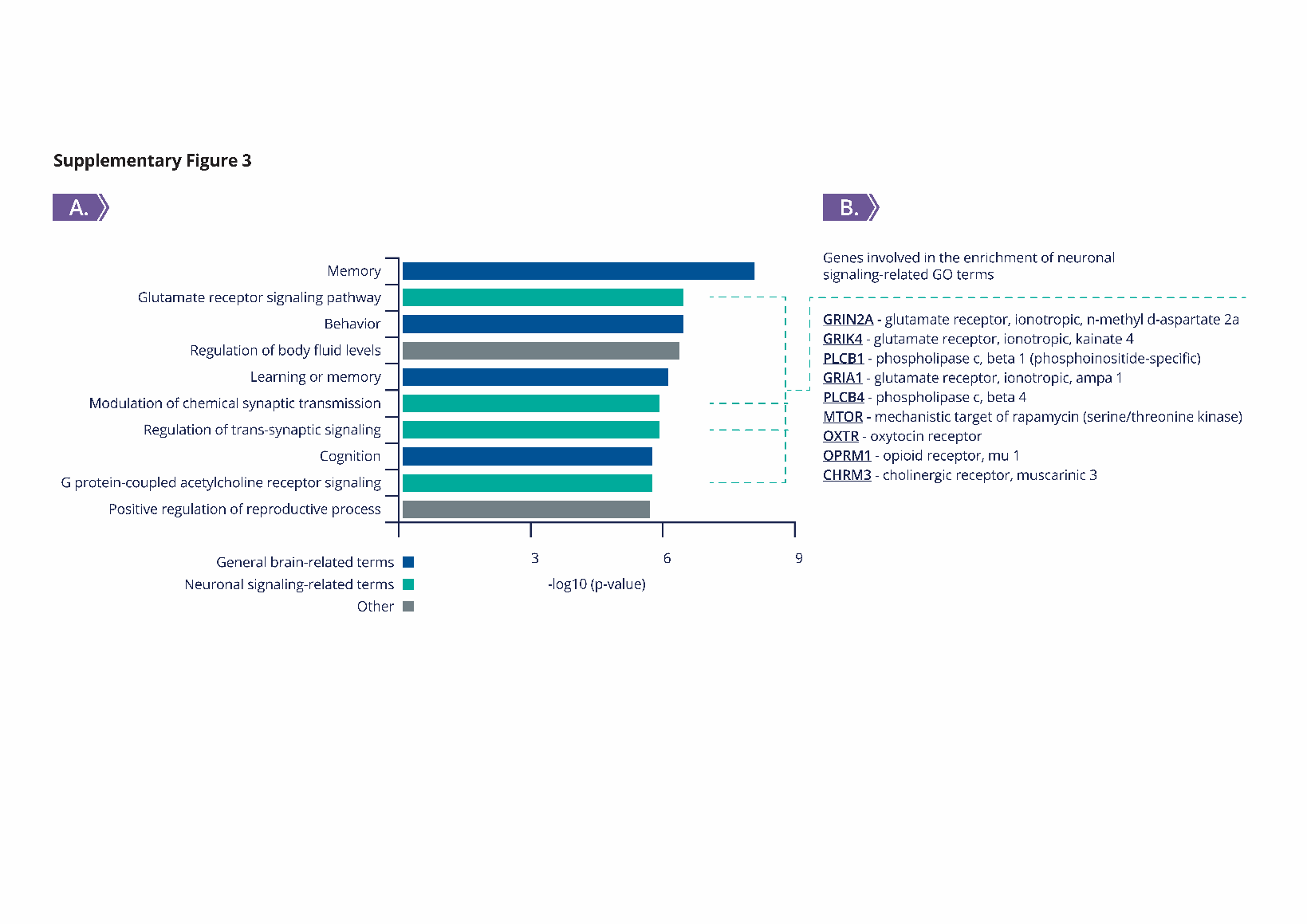
**

**References**

1. McMahon, F. J. *et al.* Variation in the Gene Encoding the Serotonin 2A Receptor Is Associated with Outcome of Antidepressant Treatment. *Am. J. Hum. Genet.* **78**, 804–814 (2006).

2. Garriock, H. A. *et al.* A Genomewide Association Study of Citalopram Response in Major Depressive Disorder. *Biol. Psychiatry* **67**, 133–138 (2010).

3. Ji, Y. *et al.* Citalopram and escitalopram plasma drug and metabolite concentrations: Genome-wide associations. *Br. J. Clin. Pharmacol.* **78**, 373–383 (2014).

4. Ji, Y. *et al.* Pharmacogenomics of selective serotonin reuptake inhibitor treatment for major depressive disorder: Genome-wide associations and functional genomics. *Pharmacogenomics J.* **13**, 456–463 (2013).

5. Gupta, M. *et al.* TSPAN5, ERICH3 and selective serotonin reuptake inhibitors in major depressive disorder: Pharmacometabolomics-informed pharmacogenomics. *Mol. Psychiatry* **21**, 1717–1725 (2016).

6. Purcell, S. *et al.* PLINK: a tool set for whole-genome association and population-based linkage analyses. *Am. J. Hum. Genet.* **81**, 559–75 (2007).

7. Browning, S. R. & Browning, B. L. Rapid and Accurate Haplotype Phasing and Missing-Data Inference for Whole-Genome Association Studies By Use of Localized Haplotype Clustering. *Am. J. Hum. Genet.* **81**, 1084–1097 (2007).

8. The 1000 Genomes Project Consortium *et al.* A global reference for human genetic variation. *Nature* **526**, 68–74 (2015).

9. The 1000 Genomes Project Consortium. An integrated map of genetic variation from 1,092 human genomes. *Nature* **491**, 56–65 (2012).

10. Templ, M., Kowarik, A., Alfons, A. & Prantner, B. VIM: Visualization and Imputation of Missing Values, Version 4.8.0. 1–69 (2019). doi:10.1007/s11634-011

11. De, R., Bush, W. S. & Moore, J. H. Bioinformatics challenges in genome-wide association studies (Gwas). *Methods Mol. Biol.* **1168**, 63–81 (2014).

12. Joiret, M., Mahachie John, J. M., Gusareva, E. S. & Van Steen, K. Confounding of linkage disequilibrium patterns in large scale DNA based gene-gene interaction studies. *BioData Min.* **12**, 11 (2019).

13. Maglogiannis, I., Karpouzis, K., Wallace, M. & Soldatos, J. *Emerging Artificial Intelligence Applications in Computer Engineering*. *IOS Press* (2007).

14. Ritter, K. *et al.* Multimodal prediction of conversion to Alzheimer’s disease based onincomplete biomarkers. *Alzheimer’s Dement. Diagnosis, Assess. Dis. Monit.* **1**, 206–215 (2015).

15. Abelaira, H. M., Réus, G. Z., Neotti, M. V. & Quevedo, J. The role of mTOR in depression and antidepressant responses. *Life Sci.* **101**, 10–14 (2014).

16. Park, S. W. *et al.* Differential effects of antidepressant drugs on mTOR signalling in rat hippocampal neurons. *Int. J. Neuropsychopharmacol.* **17**, 1831–1846 (2014).

17. Jernigan, C. S. *et al.* The mTOR signaling pathway in the prefrontal cortex is compromised in major depressive disorder. *Prog. Neuro-Psychopharmacology Biol. Psychiatry* **35**, 1774–1779 (2011).

18. Liu, X. L. *et al.* Fluoxetine regulates mTOR signalling in a region-dependent manner in depression-like mice. *Sci. Rep.* **5**, (2015).

19. Li, N. *et al.* mTOR-dependent synapse formation underlies the rapid antidepressant effects of NMDA antagonists. *Science (80-. ).* **329**, 959–964 (2010).

20. Gatt, J. M., Burton, K. L. O., Williams, L. M. & Schofield, P. R. Specific and common genes implicated across major mental disorders: A review of meta-analysis studies. *J. Psychiatr. Res.* **60**, 1–13 (2015).

21. Shelton, R. C., Hal Manier, D. & Lewis, D. A. Protein kinases A and C in post-mortem prefrontal cortex from persons with major depression and normal controls. *Int. J. Neuropsychopharmacol.* **12**, 1223–1232 (2009).

22. Bishop, J. R., Chae, S. S., Patel, S., Moline, J. & Ellingrod, V. L. Pharmacogenetics of glutamate system genes and SSRI-associated sexual dysfunction. *Psychiatry Res.* **199**, 74–76 (2012).

23. Paddock, S. *et al.* Association of GRIK4 with outcome of antidepressant treatment in the STAR*D cohort. *Am. J. Psychiatry* **164**, 1181–1188 (2007).

24. Lin, E. & Chen, P. S. Pharmacogenomics with antidepressants in the STAR*D study. *Pharmacogenomics* **9**, 935–946 (2008).

25. Whale, R. *et al.* Psychomotor retardation and vulnerability to interferon alpha induced major depressive disorder: Prospective study of a chronic hepatitis C cohort. *J. Psychosom. Res.* **79**, 640–645 (2015).

26. Lotrich, F. E. Major depression during interferon-α treatment: Vulnerability and prevention. *Dialogues Clin. Neurosci.* **11**, 417–425 (2009).

27. Antypa, N., Drago, A. & Serretti, A. Genomewide interaction and enrichment analysis on antidepressant response. *Psychol. Med.* **44**, 753–765 (2014).

28. Lekman, M. *et al.* The FKBP5-Gene in Depression and Treatment Response-an Association Study in the Sequenced Treatment Alternatives to Relieve Depression (STAR*D) Cohort. *Biol. Psychiatry* **63**, 1103–1110 (2008).

29. Binder, E. B. *et al.* Polymorphisms in FKBP5 are associated with increased recurrence of depressive episodes and rapid response to antidepressant treatment. *Nat. Genet.* **36**, 1319–1325 (2004).

30. Ising, M. *et al.* FKBP5 gene expression predicts antidepressant treatment outcome in depression. *Int. J. Mol. Sci.* **20**, (2019).

31. Tozzi, L. *et al.* Epigenetic Changes of FKBP5 as a Link Connecting Genetic and Environmental Risk Factors with Structural and Functional Brain Changes in Major Depression. *Neuropsychopharmacology* **43**, 1138–1145 (2018).

32. Hernández-Díaz, Y. *et al.* Association between FKBP5 polymorphisms and depressive disorders or suicidal behavior: A systematic review and meta-analysis study. *Psychiatry Res.* **271**, 658–668 (2019).

33. Horstmann, S. *et al.* Polymorphisms in GRIK4, HTR2A, and FKBP5 show interactive effects in predicting remission to antidepressant treatment. *Neuropsychopharmacology* **35**, 727–740 (2010).

34. Milanesi, E. *et al.* The role of GRIK4 gene in treatment-resistant depression. *Genet. Res. (Camb).* **97**, e14 (2015).

35. Scott, M. M. *et al.* Hcrtr1 and 2 signaling differentially regulates depression-like behaviors. *Behav. Brain Res.* **222**, 289–294 (2011).

36. Swann, G. *et al.* Effect of OPRM1 and stressful life events on symptoms of major depression in African American adolescents. *J. Affect. Disord.* **162**, 12–19 (2014).

37. Peciña, M. *et al.* Endogenous opioid system dysregulation in depression: implications for new therapeutic approaches. *Mol. Psychiatry* **24**, 576–587 (2019).

38. Garriock, H. A. *et al.* Association of mu-opioid receptor variants and response to citalopram treatment in major depressive disorder. *Am. J. Psychiatry* **167**, 565–573 (2010).

39. Quast, C. *et al.* Functional Coding Variants in SLC6A15, a Possible Risk Gene for Major Depression. *PLoS One* **8**, e68645 (2013).

40. Li, M., Ge, T., Feng, J. & Su, B. SLC6A15 rs1545843 and depression: Implications from brain imaging data. *Am. J. Psychiatry* **170**, 805 (2013).

41. Kohli, M. A. *et al.* The Neuronal Transporter Gene SLC6A15 Confers Risk to Major Depression. *Neuron* **70**, 252–265 (2011).

42. Je Jeon, W., Dean, B., Scarr, E. & Gibbons, A. The Role of Muscarinic Receptors in the Pathophysiology of Mood Disorders:A Potential Noveltreatment? *Curr. Neuropharmacol.* **13**, 739–749 (2015).

43. Bering, T., Carstensen, M. B., Wörtwein, G., Weikop, P. & Rath, M. F. The Circadian Oscillator of the Cerebral Cortex: Molecular, Biochemical and Behavioral Effects of Deleting the Arntl Clock Gene in Cortical Neurons. *Cereb. Cortex* **28**, 644–657 (2018).

44. Partonen, T. *et al.* Three circadian clock genes Per2, Arntl, and Npas2 contribute to winter depression. *Ann. Med.* **39**, 229–238 (2007).

45. Varinthra, P. & Liu, I. Y. Molecular basis for the association between depression and circadian rhythm. *Tzu Chi Medical Journal* **31**, 67–72 (2019).

46. Saphire-Bernstein, S., Way, B. M., Kim, H. S., Sherman, D. K. & Taylor, S. E. Oxytocin receptor gene (OXTR) is related to psychological resources. *Proc. Natl. Acad. Sci. U. S. A.* **108**, 15118–15122 (2011).

47. Noordam, R. *et al.* Identifying genetic loci associated with antidepressant drug response with drug-gene interaction models in a population-based study. *J. Psychiatr. Res.* **62**, 31–37 (2015).

48. Schneider, G. *et al.* Depressive symptoms in men aged 50 years and older and their relationship to genetic androgen receptor polymorphism and sex hormone levels in three different samples. *Am. J. Geriatr. Psychiatry* **19**, 274–283 (2011).

49. Sankar, J. S. & Hampson, E. Testosterone levels and androgen receptor gene polymorphism predict specific symptoms of depression in young men. *Gend. Med.* **9**, 232–243 (2012).

50. Hung, Huang, Chang & Kang. Deficiency in Androgen Receptor Aggravates the Depressive-Like Behaviors in Chronic Mild Stress Model of Depression. *Cells* **8**, 1021 (2019).

51. Turck, C. W. *et al.* Proteomic differences in blood plasma associated with antidepressant treatment response. *Front. Mol. Neurosci.* **10**, (2017).

52. Biernacka, J. M. *et al.* The International SSRI Pharmacogenomics Consortium (ISPC): A genome-wide association study of antidepressant treatment response. *Transl. Psychiatry* **5**, (2015).

53. Lupu, D. *et al.* Fluoxetine affects differentiation of midbrain dopaminergic neurons in vitro. *Mol. Pharmacol.* **94**, 1220–1231 (2018).

54. Kornhuber, J., Müller, C. P., Becker, K. A., Reichel, M. & Gulbins, E. The ceramide system as a novel antidepressant target. *Trends Pharmacol. Sci.* **35**, 293–304 (2014).

55. Reichel, M. *et al.* Chronic psychosocial stress in mice is associated with increased acid sphingomyelinase activity in liver and serum and with hepatic C16:0-ceramide accumulation. *Front. Psychiatry* **9**, 496 (2018).

56. Brodowicz, J., Przegaliński, E., Müller, C. P. & Filip, M. Ceramide and Its Related Neurochemical Networks as Targets for Some Brain Disorder Therapies. *Neurotox. Res.* **33**, 474–484 (2018).

57. Chekroud, A. M. *et al.* Cross-trial prediction of treatment outcome in depression: A machine learning approach. *The Lancet Psychiatry* **3**, 243–250 (2016).

58. Nasso, E. Di, Chiesa, A., Serretti, A., De Ronchi, D. & Mencacci, C. Clinical and demographic predictors of improvement during duloxetine treatment in patients with major depression: An open-label study. *Clin. Drug Investig.* **31**, 385–405 (2011).

59. Iniesta, R. *et al.* Combining clinical variables to optimize prediction of antidepressant treatment outcomes. *J. Psychiatr. Res.* **78**, 94–102 (2016).

60. Tunvirachaisakul, C. *et al.* Predictors of treatment outcome in depression in later life: A systematic review and meta-analysis. *J. Affect. Disord.* **227**, 164–182 (2018).

61. Kessler, R. C., Wai, T. C., Demler, O. & Walters, E. E. Prevalence, severity, and comorbidity of 12-month DSM-IV disorders in the National Comorbidity Survey Replication. *Arch. Gen. Psychiatry* **62**, 617–627 (2005).

62. Schneier, F. R. *et al.* Citalopram treatment of social anxiety disorder with comorbid major depression. *Depress. Anxiety* **17**, 191–196 (2003).

63. Hirschfeld, R. M. A. The comorbidity of major depression and anxiety disorders: Recognition and management in primary care. *Prim. Care Companion J. Clin. Psychiatry* **3**, 244–254 (2001).

64. Wu, Z. & Fang, Y. Comorbidity of depressive and anxiety disorders: challenges in diagnosis and assessment. *Shanghai Arch. Psychiatry* **26**, 227–231 (2014).

65. Brady, K. T., Killeen, T. K., Brewerton, T. & Lucerini, S. Comorbidity of psychiatric disorders and posttraumatic stress disorder. *J. Clin. Psychiatry* **61**, 22–32 (2000).

66. Breslau, N., Davis, G. C., Peterson, E. L. & Schultz, L. R. A second look at comorbidity in victims of trauma: The posttraumatic stress disorder-major depression connection. *Biol. Psychiatry* **48**, 902–909 (2000).

67. Leo, R. J. Movement disorders associated with the serotonin selective reuptake inhibitors. *J. Clin. Psychiatry* **57**, 449–454 (1996).

68. Perlman, K. *et al.* A systematic meta-review of predictors of antidepressant treatment outcome in major depressive disorder. *J. Affect. Disord.* **243**, 503–515 (2019).

69. Gerber, P. E. & Lynd, L. D. Selective serotonin-reuptake inhibitor-induced movement disorders. *Ann. Pharmacother.* **32**, 692–8 (1998).

70. Egger, H. L., Costello, E. J., Erkanli, A. & Angold, A. Somatic complaints and psychopathology in children and adolescents: Stomach aches, musculoskeletal pains, and headaches. *J. Am. Acad. Child Adolesc. Psychiatry* **38**, 852–860 (1999).

71. Magni, G., Moreschi, C., Rigatti-Luchini, S. & Merskey, H. Prospective study on the relationship between depressive symptoms and chronic musculoskeletal pain. *Pain* **56**, 289–297 (1994).

72. Kroenke, K. *et al.* Optimized antidepressant therapy and pain self-management in primary care patients with depression and musculoskeletal pain: A randomized controlled trial. *JAMA - J. Am. Med. Assoc.* **301**, 2099–2110 (2009).

73. del Campo, M. T., Romo, P. E., de la Hoz, R. E., Villamor, J. M. & Mahíllo-Fernández, I. Anxiety and depression predict musculoskeletal disorders in health care workers. *Arch. Environ. Occup. Heal.* **72**, 39–44 (2017).

74. Kautzky, A. *et al.* A new prediction model for evaluating treatment-resistant depression. *J. Clin. Psychiatry* **78**, 215–222 (2017).

75. Kautzky, A. *et al.* Refining prediction in treatment-resistant depression: Results of machine learning analyses in the TRD III sample. *J. Clin. Psychiatry* **79**, 16m11385 (2018).

76. Riedel, M. *et al.* Clinical predictors of response and remission in inpatients with depressive syndromes. *J. Affect. Disord.* **133**, 137–149 (2011).

77. Balestri, M. *et al.* Socio-demographic and clinical predictors of treatment resistant depression: A prospective European multicenter study. *J. Affect. Disord.* **189**, 224–232 (2016).

78. Iniesta, R. *et al.* Antidepressant drug-specific prediction of depression treatment outcomes from genetic and clinical variables. *Sci. Rep.* **8**, 1–9 (2018).

79. Uher, R. *et al.* Differential efficacy of escitalopram and nortriptyline on dimensional measures of depression. *Br. J. Psychiatry* **194**, 252–259 (2009).

80. De Carlo, V., Calati, R. & Serretti, A. Socio-demographic and clinical predictors of non-response/non-remission in treatment resistant depressed patients: A systematic review. *Psychiatry Res.* **240**, 421–430 (2016).

81. Serretti, A. *et al.* Common genetic, clinical, demographic and psychosocial predictors of response to pharmacotherapy in mood and anxiety disorders. *Int. Clin. Psychopharmacol.* **24**, 1–18 (2009).

82. Jokela, M., Lehtimäki, T. & Keltikangas-Järvinen, L. The influence of urban/rural residency on depressive symptoms is moderated by the serotonin receptor 2A gene. *Am. J. Med. Genet. Part B Neuropsychiatr. Genet.* **144**, 918–922 (2007).

83. Vigod, S. N. *et al.* Relation between place of residence and postpartum depression. *CMAJ* **185**, 1129–1135 (2013).

84. Vallée, J., Cadot, E., Roustit, C., Parizot, I. & Chauvin, P. The role of daily mobility in mental health inequalities: The interactive influence of activity space and neighbourhood of residence on depression. *Soc. Sci. Med.* **73**, 1133–1144 (2011).

85. Weaver, A., Himle, J. A., Taylor, R. J., Matusko, N. N. & Abelson, J. M. Urban vs rural residence and the prevalence of depression and mood disorder among African American women and non-Hispanic white women. *JAMA Psychiatry* **72**, 576–583 (2015).

86. Yan, X. Y., Huang, S. M., Huang, C. Q., Wu, W. H. & Qin, Y. Marital status and risk for late life depression: A meta-analysis of the published literature. *J. Int. Med. Res.* **39**, 1142–1154 (2011).

87. Bulloch, A. G. M., Williams, J. V. A., Lavorato, D. H. & Patten, S. B. The depression and marital status relationship is modified by both age and gender. *J. Affect. Disord.* **223**, 65–68 (2017).

88. Akhtar-Danesh, N. & Landeen, J. Relation between depression and sociodemographic factors. *Int. J. Ment. Health Syst.* **1**, 1–9 (2007).

89. Bjelland, I. *et al.* Does a higher educational level protect against anxiety and depression? The HUNT study. *Soc. Sci. Med.* **66**, 1334–1345 (2008).

90. Hybels, C. F. & Blazer, D. G. Epidemiology of late-life mental disorders. *Clin. Geriatr. Med.* **19**, 663–696 (2003).

91. Mirowsky, J. & Ross, C. E. Age and depression. *J. Health Soc. Behav.* **33**, (1992).

92. Mirowsky, J. Age and the Gender Gap in Depression. *J. Health Soc. Behav.* **37**, 362–380 (1996).

93. Bosworth, H. B., Hays, J. C., George, L. K. & Steffens, D. C. Psychosocial and clinical predictors of unipolar depression outcome in older adults. *Int. J. Geriatr. Psychiatry* **17**, 238–246 (2002).
